# Mechanical tests, wear simulation and wear particle analysis of carbon-based nanomultilayer coatings on Ti_6_Al_4_V alloys as hip prostheses†

**DOI:** 10.1039/c7ra12080j

**Published:** 2018-02-09

**Authors:** Ji Li, Ketao Wang, Zhongli Li, J. P. Tu, Gong Jin, Jian Su, Bao Zhai

**Affiliations:** Department of Orthopedics, General Hospital of PLA No. 28 Fuxing Road, Haidian District Beijing 100853 China lizhongli@263.net +86 010 66938306 +86 010 66938306; State Key Laboratory of Materials and Department of Materials Science and Engineering, Zhejiang University Hangzhou 310027 China; ZhongAoHuiCheng Technology Co., Economic and Technological Development Zone No. 20 Kechuang Road Beijing 100176 China; Beijing Institute of Medical Instruments No. 7 Xingguang Road, Tongzhou District Beijing 101111 China

## Abstract

Carbon-based nanomultilayer coatings were deposited on medical-grade Ti_6_Al_4_V alloy using a magnetron sputtering technique under a graded bias voltage. The mechanical properties of the nanomultilayer coatings were investigated by nanoindentation, Rockwell and scratch tests as well as a ball-on-disk tribometer. The biological properties related to the immunological response of the coatings were investigated by wear simulation and wear particles analysis. Wear simulation was done according to ISO 14242, wear particles were analysed according to ISO 17853, and then compared with CoCr femoral heads. The results revealed that the carbon-based nanomultilayer coatings showed a multilayer structure, with a hardness of ∼20 GPa, an elastic modulus of ∼175 GPa, an adhesion higher than 80 N, and a low average coefficient of friction of 0.1. The average gravimetric wear rate of the polyethylene cups between the coated and CoCr groups had no statistical difference (*P* = 0.098). The average equivalent circle diameter of particles produced in the coated group was larger than that in CoCr (*P* = 0.001), but the proportion of submicron particles and globular/circular particles was not significantly different between the two groups (*P* > 0.05). Results showed lower Co/Cr ion contamination in the coated group. Hence, the carbon-based nanomultilayer coating on Ti_6_Al_4_V has good mechanical and tribological properties, releases fewer harmful metal ions and would not cause a more intense immunological host response than a CoCr prosthesis. The newly designed a-C nanomultilayer coatings are expected to prolong the longevity of artificial hip joints.

## Abstract 2

Carbon-based nanomultilayer coatings were deposited on medical-grade Ti_6_Al_4_V alloy using a magnetron sputtering technique under a graded bias voltage.

## Graphical abstract

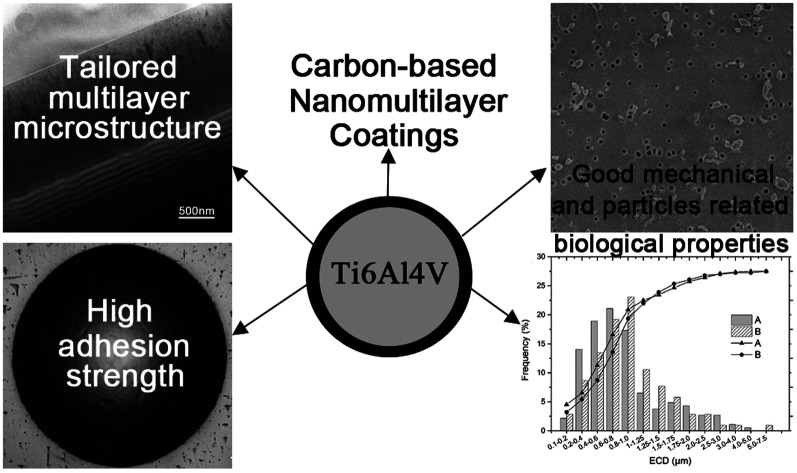

## 1. Introduction

The application of total hip arthroplasty (THA) has benefited millions of patients who suffer from severe hip joint diseases. In 2011, approximately 1.6 million THAs were conducted in 27 of the 34 member countries in the Organization for Economic Co-operation and Development (OECD).^1^ However, approximately 10–15% of patients still require revision surgery every year, and recently, the number of hip revision surgeries is rising.^2^ Thus, it is critical to increase the lifetime of hip arthroplasty. Current material standards for the artificial femoral head include cobalt chromium (CoCr) metal alloys and alumina (Al_2_O_3_) or zirconia-toughened alumina (ZTA) ceramics, which are widely used all around the world. CoCr offers a wide range of positive mechanical properties such as high strength, hardness, and elasticity. However, tribo-corrosive processes increase the levels of metal ions in local tissues and systemically,^3,4^ sometimes causing dramatic necrotic and inflammatory changes in the implant-surrounding tissue, thereby elevating the rate of bone resorption.^5–7^ For ceramics, foreign body reaction to debris is supposed to be negligible.^8^ But component fracture (incidence 0.004–2%) and squeaking (incidence 0.7–20.9%) have been reported as severe and complex problems.^9,10^.

It has been proven that wear debris, especially ultra high molecular weight polyethylene (UHMWPE) particles, can lead to deleterious biological responses, osteolysis, and ultimately component loosening and implant failure, thus decreasing the longevity of THA.^11,12^ Extensive research has been pursued to increase the biomechanical properties of prostheses for articular replacement, one solution for this problem was to coat harder materials on the articulating surfaces to improve wear resistance and reduce the generation of wear particles generated.^13–15^ Amorphous carbon (a-C) coatings such as the diamond-like carbon (DLC) is a coating produced by chemical/physical vapor deposition^16,17^ that has a low coefficient of friction, increased hardness, low wear rate and excellent biocompatibility.^18,19^ Increased wear resistance leads to the reduction in the amount of debris,^20^ allowing an increase in prostheses lifetime.^21^ However, the weak adhesion between the DLC coatings and the substrate leads to coatings chipping off, and the ultimate failure of surgeries,^22^ which is also the potential limitation for the widely use of DLC coating.^23^

To solve those problems, techniques such as bias voltage grading,^24,25^ element doping^25^ and nanomultilayer structuring^26,27^ have been applied. Our previous work indicated that the bias graded Ti-contained a-C gradient composite coating on Ti_6_Al_4_V alloy exhibited better toughness, adhesion strength and tribological performance in Hank's solution than that deposited with constant bias voltage.^24^ The adhesion problem of a-C coatings could be solved by decreasing the gradient in superficial hardness in the case of a hard coating on a relatively soft substrate. Previous literatures have suggested that nanomultilayer structures can limit crack propagation, leading to increased toughness without reducing the hardness.^14,28,29^ Therefore, in this present work, a nanomultilayer coating, which we call “a-C/a-C:Ti nanomultilayer coatings” or “carbon-based nanomultilayer coatings”, with a sequential carbon layer and carbon–Ti composite layer was deposited on medical-grade Ti_6_Al_4_V alloy using a magnetron sputtering technique under a graded bias voltage. We subsequently carried out mechanical tests, *in vitro* wear simulation and wear particles analysis to evaluate the mechanical and biological properties of the nanomultilayer coatings.

## 2. Materials and methods

### 2.1 Materials preparations

Three types of samples were prepared: Si (100) wafers were prepared to characterize coating microstructure; medical-grade Ti_6_Al_4_V alloy discs 20 mm in diameter and 2 mm in thickness were used for mechanical tests; and 28 mm diameter Ti_6_Al_4_V alloy femoral heads were used for wear simulation. The samples are shown in Fig. S1 in (ESI†). All experiments were performed in compliance with the Laboratory guidelines of Beijing Institute of Medical Instruments, and the study has been approved by the Ethics Review Board of General Hospital of PLA.

The a-C/a-C:Ti nanomultilayer coatings were deposited by a closed field unbalanced magnetron sputtering system (CC800/9 ML, CemeCon, Germany). Detailed parameters for the deposition are summarized in Table S1 of ESI.† Before the deposition, all the substrates were ultrasonically cleaned in acetone for 20 min, in ethanol for 10 min, and then blow-dried by nitrogen to clear impurities on the surface. The thicknesses of all the coatings were approximately 2 μm.

Cobalt chromium–molybdenum (CoCr) alloy femoral heads (Zimmer, America) 28 mm in diameter were used as a control group for wear simulation, all heads were articulated against UHMWPE acetabular cups (Zimmer, America, Ra: 0.8–1 μm), and eight cups were used: six test cups and two soak control cups (Table 1). The bearing surface of all acetabular cups looked polished by visual and microscope observation.

**Table 1:** Group assignment and materials of each group

Group	Femoral head	Acetabular cups	Stations number	Head Ra (μm)
A	CoCr (Zimmer)	UHMWPE (Zimmer)	Station 1, 2, 3 (s1, s2, s3)	0.013 ± 0.002
B	Ti_6_Al_4_V-coating (Jinghang)	UHMWPE (Zimmer)	Station 4, 5, 6 (s4, s5, s6)	0.016 ± 0.003
Soaking	—	UHMWPE (Zimmer)	—	—

### 2.2 Microstructure and mechanical test

Transmission electron microscopy (TEM) was used to characterize the microstructure of the coatings. A nanoindentor with a Berkovich diamond indenter (Agilent technologies, G-200, USA) was used to evaluate the hardness and Young's modulus of the coatings. The maximum indentation depth was kept at less than 10% of the coating thickness to minimize substrate effects.

To evaluate the coatings' adhesion strength, scratch tests were performed with a conventional scratch tester. A diamond pin (0.2 mm in radius) was drawn across the surface of the coating at a constant linear velocity of 4 mm min^−1^, while the load was linearly increased from 0 to 80 N. Rockwell tests were carried out using a hardness tester at a load of 100 kg using a Rockwell indenter of 0.2 mm in diameter. To evaluate the tribological properties of the coatings, a ball-on-disk tribometer with a Si_3_N_4_ ceramic ball (4 mm in diameter, hardness HV = 1550) was used. The test was performed for 60 min under a load of 5 N at a sliding velocity of 0.2 m s^−1^. The coefficient of friction (COF) was monitored continuously by a linear variable displacement transducer. The discs samples were observed under an optical microscope.

### 2.3 Wear simulation

According to ISO 14242, two groups of prosthesis couplers were studied under standard simulation conditions in a Leeds Pro Sim hip joint simulator (6 stations), with 3 samples in each group: Group A (s1, s2, s3), CoCr alloy femoral heads and Group B (s4, s5, s6), the coated Ti_6_Al_4_V femoral heads (Table 1). Before the test, the surface morphology of the femoral heads and cups were observed by SEM, the surface roughness average (Ra) of femoral heads were measured with a profilometer using a stylus radius of 2.5 μm, a scanning length of 20 mm, and a lateral resolution of 0.5 μm with a minimum of three scans per sample.

Tests were carried out in calf serum (GIBCO) (diluted with deionized water with 17% protein mass concentration) with 0.3% sodium azide to inhibit the growth of bacteria. Testing was conducted to 5 million cycles at a cyclic frequency of 1 Hz. The simulator was stopped every 500 000 cycles for serum lubricant replacement, and approximately 100 ml were collected from the test chamber at each test interval. The amount of fluid absorption was corrected by the use of soak control cups, one control cup per test, immersed in lubricant similar to that used with the test cups. During a weighing stop, the specimens and all components that had been in contact with the lubricant were carefully cleaned. Before the weighing, the cups were vacuum desiccated for 30 min. Weights were taken with a Mettler balance at a resolution of 0.01 mg. After the weighing, the specimens were reassembled, and the test was continued with fresh lubricant.

In all, there were 10 wear measurement points per test. The wear rate, expressed in mg per 1 Mc, was taken to be the slope of the regression line in the diagram showing the variation of gravimetric wear with the number of cycles. Serum samples and soaking samples were stored at −80 °C prior to particle isolation.

### 2.4 Particle isolation

The serum samples included 6 test lubricant samples from 10 testing cycles and two soak samples. Each sample was collected in an Erlenmeyer flask containing a stir bar and stirred overnight at 350 rpm. Isolation was then performed adapting a previously published standard ISO17853.^30^ Then, 10 ml of the serum sample was added to 40 ml of hydrochloric acid (37% volume fraction). The digestion was done at 50 °C for approximately 1 h until the fluid turned a slightly purple color, and then 100 ml of methanol was added to 0.5 ml of the digestion solution. The wear particles produced at each cycle were isolated and filtered under vacuum on 0.1 m Nucleopore polycarbonate filters. A make was made on the side of filter membrane containing particles.

### 2.5 Scanning electron microscope imaging (SEM)

Before the test and after 5 Mc, the surface morphology of the femoral heads and acetabular cups were observed by SEM. After drying under an infrared lamp, the filter membrane was mounted on a metal microscope stub and sputter-coated with gold at a thickness of 3 nm to 5 nm (Quorum, Q150RS). Then, the particles on the filter membrane were observed by SEM at different magnifications and SEM images of the particles were recorded. In this study, the magnification was: 500×, 1000× and 10 000× and at an accelerating voltage of no greater than 10 keV. Those parameters were adjusted according to the particle size. Semi-quantitative elementary analyses were also performed, using an Energy Dispersed Spectrometer (EDS) to determine the elements of particles. Particle morphology might be used as an additional basis for identifying UHMWPE particles by reference to published images of UHMWPE particles.^31^

### 2.6 Particles characterization and analysis

Digital images were obtained and the wear particles were characterized by image analysis, using Image-Pro Plus (Media Cybernetics) according to methods previously published.^32,33^ Evaluate the number of particles in different samples and characterize the size and shape of the particles in every image using a series of predefined descriptions such as length, width, equivalent circle diameter (ECD), area, perimeter, aspect ratio (AR), *etc.*^31^ A minimum of 20 random, nonoverlapping fields of view were analyzed for each sample.

For each filter membrane, the mean number of particles per field of view (*N*_F_) was determined, and the number of particles generated per cycle of testing (*N*_C_) was calculated as follows:

1 *N*_C_ = *N*_F_ × *A*_Filter_/*A*_Field_ × *d*/*c*

where *A*_Filter_ is the area of filter membrane, *A*_Field_ is the area of field of view, *d* is the dilution ratio, and *c* is the number of test cycles.

In this study, ECD and AR were used to characterize and compare particles in each group. ECD, a measure of particle size, is the diameter of a circle having the same area as the particle. AR is a measure of particle shape, based on the ratio of the major axis length to minor axis length. Objects with an AR in the range of 1 to 2.4 are considered to have a globular/circular shape, whereas objects with an AR in the range of 2.4 to 5 are considered to have an elongated/fibrillar shape.^34^

After drying and pressing into potassium bromide (KBr) discs, particles were examined by Fourier Transform Infrared spectroscopy (FTIR). Particles are considered to be UHMWPE if the dominant peaks in the FTIR spectra are comparable to those of a reference UHMWPE spectrum, such as the spectrum obtained from medical grade UHMWPE powder.

### 2.7 Inductively coupled plasma mass spectrometer (ICP-MS)

Samples were digested with high-purity hydrochloric acid, and the metal particles were digested into metal ions. After filtering and isolating the particles, the sample solutions were diluted with ultra-pure water and examined with the ICP-MS. The concentration of metal ions (Co, Cr, Ti and Al) were measured and recorded.

### 2.8 Statistical analysis

The SPSS 22.0 software package (SPSS, Inc., Chicago, IL, USA) was used for statistical evaluation. The Chi-square test was applied for comparisons concerning the ECD and AR proportion of submicron particles. Statistical significance was set at *P* < 0.05.

## 3. Results

### 3.1 Microstructure and mechanical properties

**Fig. 1:**
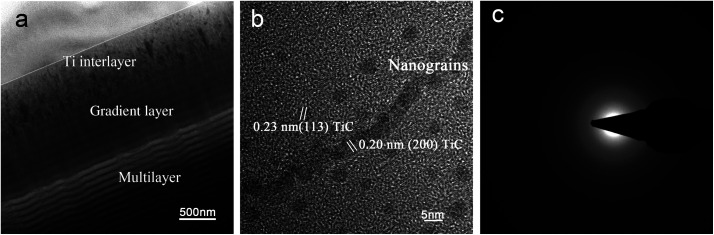
TEM characterization: (a) TEM characterization of the coatings showing multi-layer structure, (b) TEM image of nanoparticles deposited on the nanomultilayer coating, (c) selected area diffraction pattern from the coating: the absence of spots or narrow rings in the pattern indicates the crystal structure is amorphous.

The TEM characterization of a-C/a-C:Ti nanocomposite coatings showed a multilayer structure (Fig. 1) with consisting of three distinct segments: a Ti interlayer of approximately 200 nm, a C–Ti gradient interlayer of approximately 400 nm, and a-C nanomultilayer of about 1400 nm. It is demonstrated that the bilayer period of the coating was approximately 33 nm, and the thickness ratio of the a-C:Ti layer to the a-C layer was approximately 1 : 2. The interfaces between adjacent layers were moderately sharp. Unlike fine crystalline multilayer coatings, distinct interfaces are difficult to form in this a-C NM coatings. The nanomultilayer coatings deposited at constant bias voltage have similar bilayer periods and thickness ratio due to the same deposition parameters except for bias voltage. Nanocrystalline TiC embedded in a-C matrix can be found in the a-C:Ti layer, forming a nanocomposite structure, while the a-C layer exhibits an amorphous structure.

**Fig. 2:**
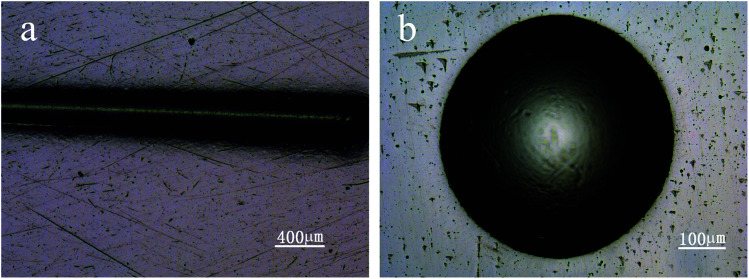
Optical images of scratch track (a), Rockwell crater (b) of carbon-based nanomultilayer coatings.

Nanoindentor showed that the coating had a hardness of ∼20 GPa and an elastic modulus of ∼175 GPa. The *H*_3_/*E*_2_ and *H*/*E* values of the coatings are 0.262 and 0.120. According to previous work,^26,35,36^*H*_3_/*E*_2_ and *H*/*E* are suggested as an indicator of toughness, namely the resistance to plastic deformation and wear resistance, respectively. The coatings with high *H*_3_/*E*_2_ and *H*/*E* values indicate that nanomultilayer deposited under graded bias voltage may display high toughness. Adhesion strength of the coatings was measured using scratch and Rockwell tests. Fig. 2a shows the scratch traces on the bias-graded nanomultilayer coatings. Scratch tests showed that the critical load of the coating was higher than 80 N and there was no obvious fragment or delamination in the whole scratch trace. Rockwell tests showed that no evident crack and coating delamination could be observed under a load of 100 kg (Fig. 2b). The ball-on-disk test at an applied load of 5 N showed the coatings with a graded bias had a low average COF of 0.1.

### 3.2 Wear simulation

#### 3.2.1 Wear rate

The average gravimetric wear rate of polyethylene cups against the Ti-coated heads was higher than that of the CoCr heads (35.23 ± 1.31 *vs.* 32.65 ± 1.08 mg per 10^6^ cycles), but there was no statistical difference between the two groups (*t* = −3.352, *P* = 0.076). The total weight gains of the soak control cups, 0.9–2.3 mg, were small compared with the total weight losses of the test cups, which were 98–136 mg.

#### 3.2.2 Surface morphology

**Fig. 3:**
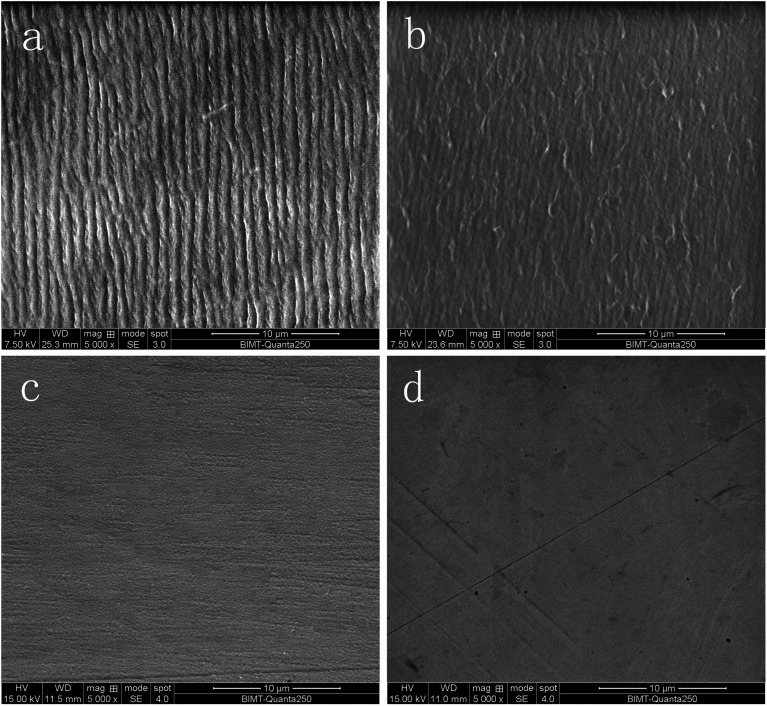
Surface morphology after 5 Mc: (a) s1 cup, (b) s4 cup, (c) s1 femoral head, (d) s4 femoral head.

After 5 Mc of *in vitro* wear simulation, all heads proved to be practically unchanged by visual and microscope observation, and there was no chipping of the coatings (Fig. 3a and b). All heads showed the same texture as before the wear simulation, and the surface roughness value Ra was unchanged.

After articulation against femoral heads, the bearing surface of all acetabular cups still looked polished by visual and microscope observation. There were no obvious signs of wear on the outer surface of the cups. The striation feature on the bearing surface of the cups revealed that the wear mechanism was adhesive (Fig. 3c and d).

#### 3.2.3 ICP-MS

The analysis of ICP-MS shows that Ti/Al and Co/Cr ions were found in the lubricant. Table 2 showed the contamination of these metal ions in two groups, and it is obvious that the level of Co/Cr ions in Group A were higher, but the level of Ti/Al ions were not significant differences between two groups.

**Table 2:** The contamination of different metal ions in two groups

	Group A	Group B	*t*	*P*
Co (μg L^−1^)	3.12 ± 0.14	0.57 ± 0.07	27.573	0.000
Cr (μg L^−1^)	2.73 ± 0.71	0.47 ± 0.28	6.082	0.040
Ti (μg L^−1^)	0.96 ± 0.41	1.05 ± 0.54	−2.362	0.077
Al (μg L^−1^)	1.06 ± 0.39	1.23 ± 0.35	−2.643	0.057

### 3.3 Wear particles characterization and analysis

**Fig. 4:**
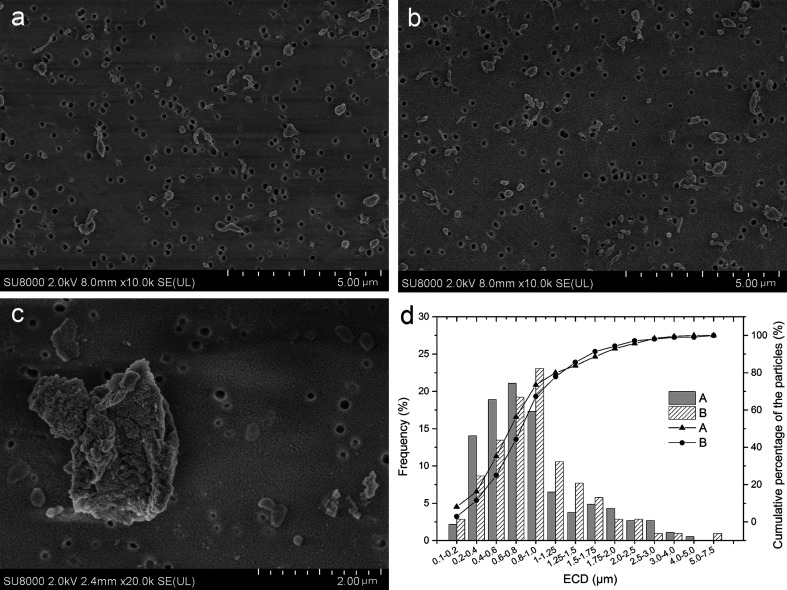
Particles distribution under SEM: (a) particles distribution of s1 (b) particles distribution of s4, (c) large flakes from s1 have a more complex surface morphology than small particles, (d) particle size distribution of UHMWPE particles and the cumulative percentage of different size particles in the lubricant of CoCr (Group A) and coated-Ti (Group B). In both groups, the majority of particles had an ECD of less than 1 μm. The mean ECD (standard error) was 0.516 ± 0.408 and 0.627 ± 0.667 for Group A and B (*P* = 0.001), and the proportion of submicron particles was 73.51% and 67.31% in Group A and B, respectively (*P* = 0.163).

The distribution and micro morphology of particles are shown in Fig. 4. By SEM, we can see that particles were dispersed and almost none overlapped, and just a few particles gathered. In both groups, the wear particles isolated from the serum lubricants were mainly submicron- and micron-sized (Fig. 4a and b), but a very small number of larger flakes were also observed (Fig. 4c). The surfaces of the smaller particles were smooth, regular and tended to roundness (Fig. 4a and b), while the surfaces of large flakes were more rough and complex (Fig. 4c). There were almost no particles in the soak control group.

EDS results showed that the overwhelming element was C, which is the major component of polyethylene, while O may come from the polycarbonate filter coating (Fig. S2†). Additionally, a few Au sputter-coated particles were observed under SEM. Particle morphology was observed to be similar to the published images of UHMWPE particles. FTIR spectrums of the particles in two groups were similar to the reported spectrum of UHMWPE (Fig. S3†).

The test interval (in cycles) for each serum sample is listed in Table 3. Table 3 showed that the average particle count per cycle of lubricant was slightly higher in the CoCr than the coated-Ti (5.93 × 10^6^ ± 0.55 × 10^6^*vs.* 5.36 × 10^6^ ± 0.33 × 10^6^ per cycle), but no difference was found between them (*t* = 2.183, *P* = 0.054). Fig. 4d shows the average ECD was 0.475 ± 0.419 and 0.704 ± 0.694 μm in Group A and B, respectively (*t* = −3.501, *P* = 0.001), meaning that the particles in Group A are larger than Group B. The majority of the polyethylene wear particles detected on the filters were in the 0.1–1 μm (submicron) size range, and the proportion of submicron particles in Group A was higher than that in Group B (73.51% *vs.* 67.31%), though there were no statistical differences (*χ*^2^ = 1.252, *P* = 0.163).

**Table 3:** Data on serum samples harvested from hip simulator

Serum samples	Test interval (MC)	*N*_F_ a		*N*_c_ b × 10^6^	
		Group A	Group B	Group A	Group B
1	0–0.5	71.9	68.3	5.82	5.53
2	0.5–1	76.5	67.9	6.19	5.50
3	2–2.5	82.4	62.5	6.67	5.06
4	3–3.5	76.6	71.4	6.20	5.78
5	3.5–4	63.1	60.4	5.11	4.89
6	4.5–5	68.7	66.3	5.56	5.37
Average		73.2	66.1	5.93	5.36
S.D.		6.80	4.04	0.55	0.33

a Mean number of particles per field of view (10 000× magnification).

b Mean number of particles generated per cycle.

**Fig. 5:**
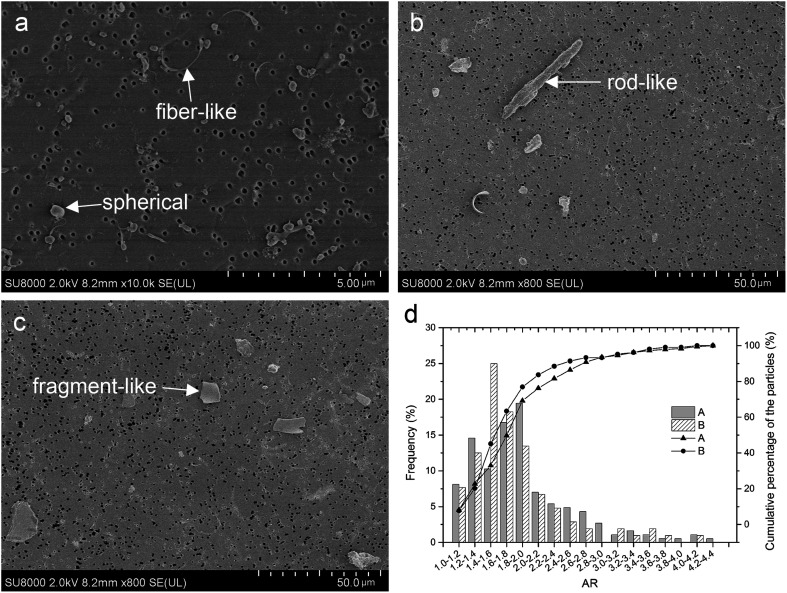
Different particles shapes: (a) spherical and fiber-like, (b) rodlike, (c) fragment-like, (d) the shape distribution and the cumulative percentage of spherical particles in two groups. (d) Particle shape distribution of UHMWPE particles and the cumulative percentage of different shape particles in lubricant CoCr (Group A) and coated-Ti (Group B). The majority of the polyethylene wear particles were globular/circular (AR ≤ 2.4) in both groups, the proportion of submicron particles was 88.46% and 81.62% in Group A and B, respectively (*P* = 0.085).

Many different shapes of particles are described in Fig. 5a–c. Fig. 5d shows that 88.46% in Group A and 81.62% in Group B of the polyethylene wear particles detected on the filters have ARs less than 2.4, meaning that majority of the particles were globular/circular in both groups, but the proportion of globular/circular particles between the two groups was not significantly difference (*χ*^2^ = 2.327, *P* = 0.085).

## 4. Discussion

The wear of bearing surfaces has been cited as a dominant factor limiting the longevity of implants.^37^ Modern demographic developments and lifestyles are posing dramatic challenges for implant designs and materials. In this present work, a bias-graded multilayer structure coating with sequential carbon and the carbon–Ti composite layer was deposited on medical-grade Ti_6_Al_4_V alloy using unbalanced magnetron sputtering under graded bias voltage,^38,39^ which can maintain high levels of ion bombardment during deposition and is a well-established technique for the preparation of industrially hard coatings.^40^ The multilayer structure can deflect or reduce crack propagation and decrease stress concentration. Additionally, a bias-graded coating is an effective method to achieve increased hardness and toughness as well as high adhesion strength.^24^ As the coating thickness increases, the coating hardness will decrease. Thus the coating is not the thicker the better. Hauert *et al.* suggested that a coating used in bio-tribological applications should be more than 1 μm thick.^13^ Based on our practical applications and tests, and to match other parts of the artificial hip joint, the ideal coating thickness is generally 2–3 μm. The results in this study show that the nanomultilayer coating on Ti_6_Al_4_V alloy has an appropriate hardness of 20 GPa, increased toughness and high adhesion strength of approximately 80 N, solving the problem of weak adhesion strength in traditional DLC coatings. The use of C–Ti gradient interlayer could help to decrease the high gradient in superficial hardness and improve the adhesion between Ti_6_Al_4_V and a-C coating. Additionally, the enhanced hardness is mainly ascribed to the presence of nanocrystalline TiC and plenty of interfaces, which can hinder dislocation movements. Moreover, the improved adhesion strength of the coating results from the bias-graded structure which ensures no abrupt change in composition and high toughness as above-mentioned. Compared to a previous work,^33^ the adhesion strength of bias-graded nanomultilayer is obviously higher than that of bias-graded nanocomposite coatings, which may result from nanomultilayer structure in which the interfaces become the sites of energy dissipation and crack deflection. Additionally, the coating exhibits low friction and high wear resistance due to a graphite-like trilayer. Our previous studies showed that the sp^2^ and sp^3^ structure of the nanomultilayer coating determines the high hardness and self-lubricating characteristic, and the self-lubrication characteristic of the graphitic coating helps the steady COF.^24,28^ The nanomultilayer coatings have a low average COF of 0.1, and it is a lower value compared with other coating such as DLC in literature,^41,42^ but the COF may be affected by the testing conditions in different studies. Thus, microstructure and mechanical tests showed that the carbon-based nanomultilayer coatings have desirable mechanical and tribological properties.

A wear simulation study was done with a hip wear simulator using the fetal bovine serum as a lubricant with one million cycles in a simulator generally taken to represent 1 year *in vivo*. After 5 Mc of hip simulation, the average gravimetric wear rate of polyethylene cups against Ti-coated heads was no different with that against CoCr heads (*P* = 0.098). Studies have reported that in more than 70% of all THA revisions, implant aseptic loosening has proved to be the major reason for premature failure and is mainly associated with the biological potential of wear debris,^6^ especially UHMWPE debris.^12,43,44^ The role of UHMWPE particles in immunologic response and ultimately osteolysis is a complex cascade involving multiple cell types.^45^ In this study, we used EDS to determine the elements of the particles and found that the overwhelming element were C, which is the major components of polyethylene, O may come from the polycarbonate filter coating. Although the surface coating consisted of C, no obvious chipping of the coatings was found, so the particles were not from the coating. Additionally, the FTIR results were similar to the reported spectrum of UHMWPE, and the observed particle morphology was similar to the published images of UHMWPE particles. Thus we can infer that the particles isolated from the lubricant are UHMWPE.

It has been demonstrated that many characteristics of the particulate debris especially the number, size, and shape, can influence the severity of cellular response.^46–49^ The authors considered that the immunological response of the host is more intense for particles with smaller sizes, including a greater release of the tumor necrosis factor cytotoxin.^50–54^ Studies showed that particles within submicron to 10 μm range may be the most provocative in the pathogenesis of aseptic loosening, because particles in this size range were readily engulfed by polymorph nuclear cells.^50,51^ In particular, submicron wear particles have been implicated as a potentially important contributor to the onset of osteolysis.^55,56^ Tipper *et al.* characterized debris from UHMWPE against zirconia ceramic and found that approximately 85% of the debris had a size of 0.1–0.5 μm^57^. Wu and Peng studied debris from UHMWPE against CoCr alloys and found that approximately 71.6% were in a size range of 0.1–10 μm.^58^ In this study, the average particle number per cycle of lubricant between two groups was no difference, particles produced by coated heads were larger than that produced by CoCr, but the proportion of submicron particles between two groups was no statistical differences. Furthermore, the authors have shown that the shape of patient-derived wear debris has a strong influence on the severity of the tissue inflammatory response. Debris with a fibular shape incited a substantially higher IL-1 and TNF expression when compared to debris with a smooth, globular shape.^34,59^ AR was used to measure the particle shape in our study, and we found that the proportion of globular/circular shape particles (1 ≤ AR ≤ 2.4) between two groups was also not significantly different. Therefore, we can infer that, compared with CoCr, particles produced by the Ti_6_Al_4_V with carbon-based nanomultilayer coating would not make more intense immunological host response.

Minute quantities of Ti/Al and Co/Cr ions existed in normal human and animal body, but too much is toxic. Increased Co/Cr ions in local tissues and systemically may cause dramatic necrotic and inflammatory changes in the implant-surrounding tissue, thereby elevating the rate of bone resorption. Thus minimizing Co/Cr ions concentration is very important to prolong the longevity of prostheses. Ti_6_Al_4_V is a kind of metal with good biocompatibility, and it has been widely used as orthopedic implants. However, Ti_6_Al_4_V cannot be used as the bearing surface of the artificial joint directly due to its relatively low hardness. The carbon-based nanomultilayer coating improves the hardness of Ti_6_V_4_V surface and could act as an ion barrier to minimize the release of ions due to tribo-corrosion and three-body abrasion, additionally, the chemical composition and structure of the coating are biological inert, avoiding the host biological response.^10,11^ Results of ICP-MS showed that compared with CoCr heads, less Co/Cr ions were found in the Ti-coated group, but the level of Ti/Al ions were not significantly different between two groups. Additionally, in clinic, use the coated Ti_6_Al_4_V head could unify the metal material of femoral head and stem, which could also decrease the corrosion in the taper joint caused by potential difference of different material.

## 5. Conclusion

The carbon-based nanomultilayer coatings on Ti_6_Al_4_V have good mechanical and tribological properties, release fewer harmful metal ions and would not create a more intense immunological host response compared with a CoCr prosthesis. The newly designed a-C nanomultilayer coatings are expected to prolong the longevity of artificial hip joint.

## Conflicts of interest

There are no conflicts to declare.

## Supplementary Material

RA-008-C7RA12080J-s001
