# Supplementary material for: Mechanical tests, wear simulation and wear particle analysis of carbon-based nanomultilayer coatings on Ti6Al4V alloys as hip prostheses
Source: RSC Adv. 2018 Feb 9;8(13):6849–57. doi: 10.1039/c7ra12080j (PMC9078386; doi:10.1039/c7ra12080j)
Supplement: RA-008-C7RA12080J-s001 [file RA-008-C7RA12080J-s001.pdf]

Electronic Supplementary Material (ESI) for RSC Advances.
This journal is © The Royal Society of Chemistry 2018

Supplementary Information

Mechanical tests, wear simulation and wear particles analysis of carbon-based nanomultilayer coatings on Ti6Al4V alloys as hip prosthesis

Ji Li[a]†, Ketao Wang[a]†, Zhongli Li[a]*, J. P. Tu[b], Gong Jin[c], Jian Su[d], Bao Zhai[a]

a. Department of Orthopedics, General Hospital of PLA, Beijing 100853, China

b. State Key Laboratory of Materials and Department of Materials Science and Engineering, Zhejiang University, Hangzhou 310027, China

c. ZhongAoHuiCheng Technology Co. Ltd, Beijing 100176, China.

d. d. Beijing Institute of Medical Instruments, No.7 Xingguang Road, Tongzhou District, Beijing 101111, China.

---

†These authors contributed equally to this work.

* Corresponding authors. Tel.: +86 010 66938306; fax: +86 010 66938306;

E-mail addresses: lizhongli@263.net;

Table S1. Depositing parameters for graded layer of a-C/a-C:Ti nanomultilayer films

Process	Substrate clean	Target clean	Interlayer	Transition layer	Nanomultilayer
Time (min)	30	5	10	60	130
Substrate bias (V)	-500	-500	-200	-100	-50, -100, -150, -50 → -150
Ti target current (A)	0.2	0.2	2.5	2.5→1	1
C target current (A)	0	0.2	0.2	0.2→2.5	2.5
Degree of vacuum (Pa)	0.2	0.2	0.2	0.2	0.2
Substrate holder rotation	no	no	no	yes	y

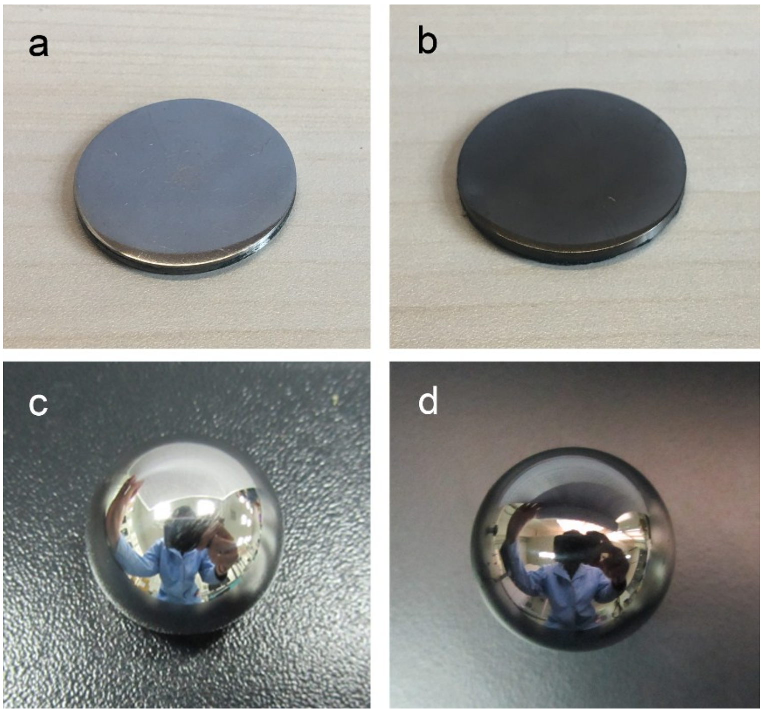

Fig.S1 (a) Ti6Al4V disc (b) Ti6Al4V disc with the carbon based nanomultilayer coatings (c) CoCr alloy femoral head (Zimmer, America), (d) Ti6Al4V femoral head (Jing hang, China) with a-C/aC:Ti nanomultilayer coatings.

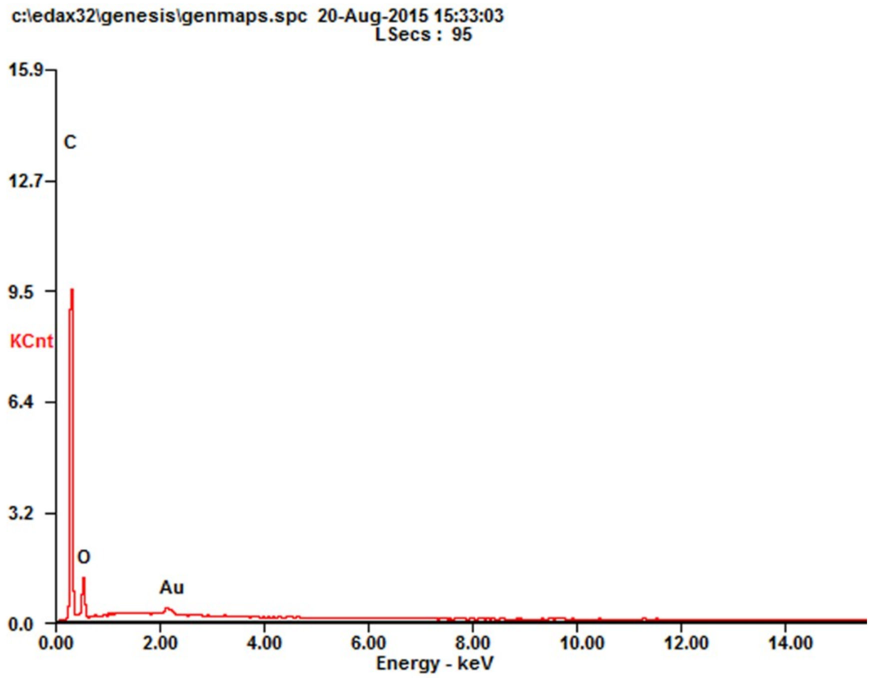

Fig. S2 EDS results showed that the overwhelming element was C, which is the major component of polyethylene, while O may come from the polycarbonate filter coating. Additionally, a few Au sputter-coated particles were observed under SEM

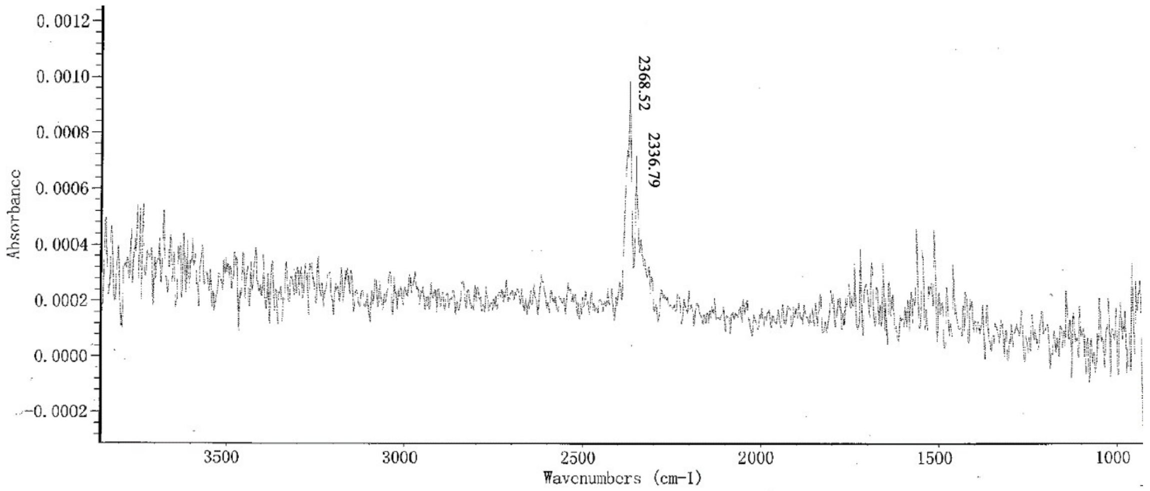

Fig. S3 FTIR spectrum of the particles were similar to the reported spectrum of UHMWPE.
